# Clinical utility of serum biomarkers in Duchenne muscular dystrophy

**DOI:** 10.1186/s12014-016-9109-x

**Published:** 2016-04-05

**Authors:** Yetrib Hathout, Haeri Seol, Meng Hsuan J. Han, Aiping Zhang, Kristy J. Brown, Eric P. Hoffman

**Affiliations:** Center for Genetic Medicine, Children’s National Healthy System, Washington, DC USA

**Keywords:** Duchenne muscular dystrophy, Biomarkers, miRNA, Proteins, Pharmacodynamic biomarkers, Surrogate biomarkers, Clinical outcomes, Mass spectrometry, SomaScan

## Abstract

Assessments of disease progression and response to therapies in Duchenne muscular dystrophy (DMD) patients remain challenging. Current DMD patient assessments include complex physical tests and invasive procedures such as muscle biopsies, which are not suitable for young children. Defining alternative, less invasive and objective outcome measures to assess disease progression and response to therapy will aid drug development and clinical trials in DMD. In this review we highlight advances in development of non-invasive blood circulating biomarkers as a means to assess disease progression and response to therapies in DMD.

**Electronic supplementary material:**

The online version of this article (doi:10.1186/s12014-016-9109-x) contains supplementary material, which is available to authorized users.

## Background

 Duchenne muscular dystrophy (DMD) is a severe form of myopathy that affects 1 in 5000–20,000 male births worldwide [1, 2]. It is due to frame shift mutations in the X-linked *dystrophin* gene abolishing the expression of the dystrophin protein [3]. Dystrophin connects the muscle fiber cytoskeleton to the extracellular matrix environment through the dystrophin associated complex (DAPC), a structural complex that protects the sarcolemma from injuries during muscle fiber contraction and relaxation [4, 5]. Absence of dystrophin results in the disturbance of the DAPC leading to micro-tears in the sarcolemma and calcium influx followed by muscle fiber deterioration and tissue inflammation. Clinically, DMD is characterized by progressive muscle necrosis and wasting leading to loss of ambulation by 8–12 years of age and death by early adulthood due to cardiorespiratory failure [6]. Currently there is no cure for the disease only treatment to reduce muscle inflammation with glucocorticoids such prednisone and deflazacort. This treatment delays loss of ambulation by 1–2 years, but with no long term benefit and often accompanied by debilitating side effects [7–9]. Recently, multiple drug development programs focusing on slowing or preventing the progressive muscle pathogenesis in DMD have emerged [10]. These included therapies aiming to restore expression of the missing dystrophin [11–15] and a new corticosteroid dissociative drug with potential for greater anti-inflammatory benefits and fewer side effects [16]. However, lack of reliable outcome measures to assess efficacy of these drugs had delayed approvals from regulatory agencies. So far only the PTC Therapeutics’ Translarna (ataluren) drug, a stop codons read through drug, has received a conditional approval from the European Medicines Agency (EMA) to treat specific group of DMD patients with nonsense mutations in the *dystrohin* gene [17, 18].

## Current outcome measures and challenges in DMD

Establishing disease-appropriate outcome measures for clinical trials in neuromuscular disorders has been challenging. As for any pediatric degenerative disease it is critical that outcome measures be specific and sensitive to disease progression and response to treatment. However, this becomes a challenge when using a small number of pediatric patients and when the therapeutic approach may be of high risk and the treatment is administered for a short period of time.

Current standardized outcome measures used in DMD clinical trials include the 6 min walk (distance patient can walk in 6 min) [19], the North Star Ambulatory assessment consisting of number of physical and observational test [20], as well as quantitative muscle strength tests [21]. However, lack of cooperation in young patients, as well as inclusion of ambulant patients only, limits the above testing to a subset of the DMD population. Furthermore, it might take longer to observe clinical benefit using these physical tests.

Magnetic resonance imaging and T2 mapping are a less invasive approach to monitor disease progression and response to treatment in DMD patients [22, 23]. While these imaging techniques are useful in monitoring muscle loss, cardiac function and other vital parts in DMD patients they are often costly, laborious and low throughput (e.g. 1–2 h machine time per patient).

Molecular biomarkers measured in blood could prove valuable to assess disease progression and response to therapies in DMD boys. Biomarkers as applied to a given disease such as DMD, have utility in one or more of the following areas: (1) they are easily accessible and less invasive to patients, (2) can act as a diagnostic tool (a panel of biomarkers is more specific and reliable than a single biomarker); (3) can act as pharmacodynamic biomarkers to monitor safety and efficacy of a new treatment; (4) can act as surrogate biomarkers to anticipate or predict later clinical benefit of an intervention; (5) can inform disease pathophysiological pathways that can lead to development of novel therapeutic targets [24].

This review focuses on the latest advances in molecular biomarker development for DMD with emphasis on serum protein biomarkers and their relationship with disease progression and response to therapy.

## Recent biomarker developments for DMD

Over the past 5 years a series of international meetings and workshops have identified the critical need for and dissemination of a reliable molecular marker to monitor DMD disease progression and response to treatment [25]. This need has become urgent as promising therapeutic strategies are entering clinical trials [10]. A current challenge in monitoring response to therapies in DMD is that different outcome measures are required at different stages of the disease, and these measures can sometimes be subjective and lack sensitivity. For example, the 6 min walk test, the most common outcome measure in DMD clinical trials, can only be applied to ambulant DMD patients (≥5 years of age) and requires a large cohort and a long time after treatment to detect a meaningful outcome. Additionally, very young patients (≤4 years of age) and non-ambulant patients (≥12 years of age) are often excluded from clinical trials due to lack of reliable outcome measures for these specific age ranges. In this context molecular biomarkers are expected to be less subjective, more robust and could be implemented throughout disease stages and patient ages. Biomarkers could help provide evidence of drug efficacy in acute time frames (pharmacodynamic biomarkers) as well as predict later clinical outcomes (surrogate biomarkers). The need of such biomarkers in DMD is further supported by the recent regulatory status of eteplirsen (a dystrophin replacement drug for DMD) highlighting the importance of dystrophin restoration as surrogate biomarker to predict benefit (see recent FDA Briefing Document, UCM481911, 2016). The importance of accurate measurement of levels of restored dystrophin in muscle biopsies and the poor precision of Western blot were also highlighted in this report and in a review article [26]. In this context our laboratory has developed a targeted mass spectrometry assay to accurately quantify dystrophin in muscle biopsies [27–29]. The method was found to be highly reproducible and linear over wide dynamic range with a precision <20 % CV. Dystrophin measurement is an ideal outcome measure in clinical trials using dystrophin replacement therapies but requires multiples muscle biopsies, which is a burden for pediatric patients. Biomarkers accessible in body fluids, on the other hand, are less invasive and might prove useful for predicting treatment efficacy and outcomes.

Different types of molecular biomarkers have been identified in serum, plasma and/or urine of DMD patients. These included metabolites, miRNA and proteins. While miRNAs and proteins have been well developed in the past 5 years, only few studies have been published on metabolite biomarkers in DMD [30]. In this review we will focus mostly on recent development of miRNA and protein as biomarkers for DMD.

### Blood circulating miRNAs as biomarkers for DMD

One of the earliest studies investigating miRNA as potential biomarkers for DMD was based on the potential leakge of muscle specific miRNA (dystromiRs) into the blood stream of DMD patients [31]. Three miRNA biomarkers (miR-1, miR-133, and miR-20) were identified and found highly elevated in serum of DMD patients and patients with Becker’s muscular dystrophy (BMD), a less severe form of dystrophinopathy, compared to healthy controls. These same miRNAs were also identified as elevated in the mdx mouse model for DMD, and their levels decreased when dystrophin was restored by exon skipping therapy [31]. After this initial study, a series of studies conducted in the mdx mouse model and DMD patients demonstrated the utility of circulating miRNA as biomarkers to monitor disease severity and response to treatments aiming to restore the missing dystrophin protein [32–34]. In a recent study, using high-throughput miRNA sequencing, additional miRNAs including cardiomyopathy related miRNAs were identified in the golden retriever muscular dystrophy dog model and further confirmed in DMD patients [35]. Overall, studies in both animal models and patients identified the same set of candidate serum circulating miRNAs. This suggests the robustness and the specificity of these miRNAs as biomarkers for dystrophinopathies. Interestingly, most of the elevated circulating miRNAs in serum of DMD and mdx mouse model were found unchanged or even decreased in muscle tissue.

### Serum circulating proteins as biomarkers for DMD

Initial protein biomarker studies in DMD used ELISA and other enzyme linked assays to target specific proteins based on their relation with muscle damage and pathogenesis. These included creatine kinase M (CK-M), a muscle specific protein that reflects sarcolemma damage. CK-M was reported along ago to be elevated in serum of DMD patients relative to controls [36] and it is currently used to screen for DMD in newborns [37]. Although CK is a good marker to screen for suspected dystrophinopathies it is not suitable to monitor disease progression and response to therapy because it decreases sharply with age and its concentration is easily influenced by muscle trauma and exercise. Although, recent drisapersen trial, have shown the utility of CK as a secondary endpoint to assess efficacy [25].

Carbonic anhydrase III (CA-III) and myoglobin are other two muscle specific proteins that were targeted for analysis by ELISA and were found elevated in blood of DMD patients [38, 39]. MMP9, TIMP1 and osteopontin are associated with muscle inflammation and were also targeted for analysis by ELISA and had altered levels in serum of DMD patients relative to healthy controls [40].

More recently, high throughput “omic” approaches were introduced to survey hundreds to thousands of proteins simultaneously in serum samples of DMD patients. These techniques confirmed the previously identified biomarkers, and also uncovered novel markers that are associated with muscle pathogenesis and disease progression.

A study by Ayoglu et al. [41] used an affinity proteomics approach and identified 11 serum protein biomarkers associated with DMD, of which 5 were novel and included myosin light chain-3 (MYL3), troponin T, fast skeletal muscle (TNNT3), plastin-2 (LCP1), protein phosphatase 1F (PPM1F) and electron transfer flavoprotein A (ETFA). In this study the authors used bead arrays consisting of up to 384 antibodies selected from the Human Protein Atlas repository that were directed against 315 different proteins and screened a total of 345 plasma/serum samples from muscular dystrophy patients. The method was found to be sensitive with highly multiplexing and throughput capabilities.

During the same time period, our lab used two high throughput technologies, mass spectrometry based proteomics profiling and SomaScan aptamer array approach [42, 43], to query about 1500 unique proteins in serum samples of DMD patients. These were performed across the age range of DMD patients (4–29 years old). In the initial mass spectrometry based proteomics approach we queried about 300–400 serum proteins across the age range in both DMD patients and two independent mdx models for DMD and identified 21 protein biomarkers that were concordant between the mouse models studied and DMD patients [44]. These biomarkers were mostly of muscle origin and included myofibrillar proteins (titin, myosin light chain 1/3, myomesin 3, filamin-C), glycolytic enzymes (aldolase, phosphoglycerate mutase 2, beta enolase, and glycogen phosphorylase), transport proteins (fatty acid binding protein-3, myoglobin and somatic cytochrome-C), and others (creatine kinase M, metalloproteinase-9, malate dehydrogenase cytosolic and fibrinogen gamma). In the second discovery method, we used highly multiplexing and high throughput SomaScan aptamer technology to query 1129 unique proteins in serum of DMD patients (n = 93) and age matched healthy volunteers (n = 45) across the age range or 4–29 years old. In this second discovery study we identified 44 robust serum biomarkers that were concordant between two independent cohorts [45].

**Fig. 1:**
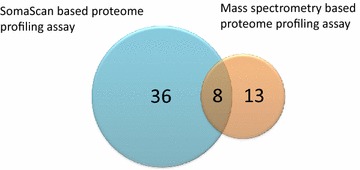
Venn diagram showing overlapping and unique number of identified biomarkers by SomaScan and mass spectrometry based proteome profiling techniques. Both techniques combined identified and quantified 59 biomarkers in DMD sera samples. Biomarkers that were uniquely identified by mass spectrometry are mostly myofibrillar proteins for which there was no Somamer designed. Biomarkers that were uniquely identified by SomaScan are those that are below mass spectrometry detection limit

Overall using a combination of mass spectrometry based proteomics and SomaScan aptamer array we identified 59 protein biomarkers associated with dystrophin deficiency in serum of DMD patients (see Additional file 1: Table S1). There were strengths and weaknesses of both platforms. The SomaScan required only a small sample volume (65 μl per assay), is sensitive, linear over wide dynamic range and did not require albumin depletion, but has a poor coverage of myofibrillar proteins and other protein fragments (e.g. titin, myomesin-3, filamin C, actin skeletal muscle type and myosin light chain 1/3). The mass spectrometry based proteomics profiling approach is considerably more labor intensive and required protein fractionation to separate albumin from low abundant proteins, but has detected several muscle-derived biomarkers including myofibrillar proteins and other proteins that SomaScan technology did not detect because there is no aptamer designed for this class of proteins. Nevertheless, Serum proteome profiling using SomaScan technology remains superior to mass spectrometry based approach in term of sensitivity, dynamic range and throughput. Figure 1 shows the overlap and differences in the number of biomarkers detected by the two techniques.

In summary, using protein affinity arrays and mass spectrometry based proteomics a comprehensive list of serum protein biomarkers has been established and is now publically available to researchers in the field of muscular dystrophies for data mining and testing (see Additional file 1: Table S1).

## Circulating biomarkers are indicative of early stage muscle damage in DMD boys

**Fig. 2:**
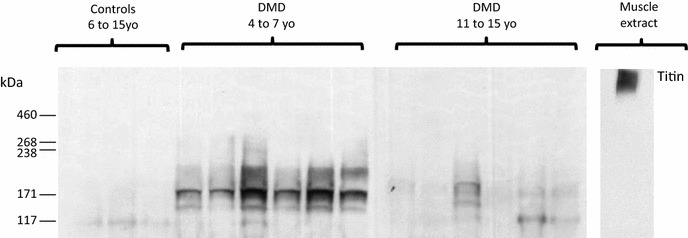
Western blot showing detection of titin fragment in serum of DMD patients. Titin was detected as a fragment around 170 kDa in serum of young DMD patients but very low in serum of older DMD patients and healthy controls. A positive control showing detection of full length titin in human muscle extract is also shown. Note that full lenght titin is 3 mega Dalton protein and stayed in the well of the gel

**Fig. 3:**
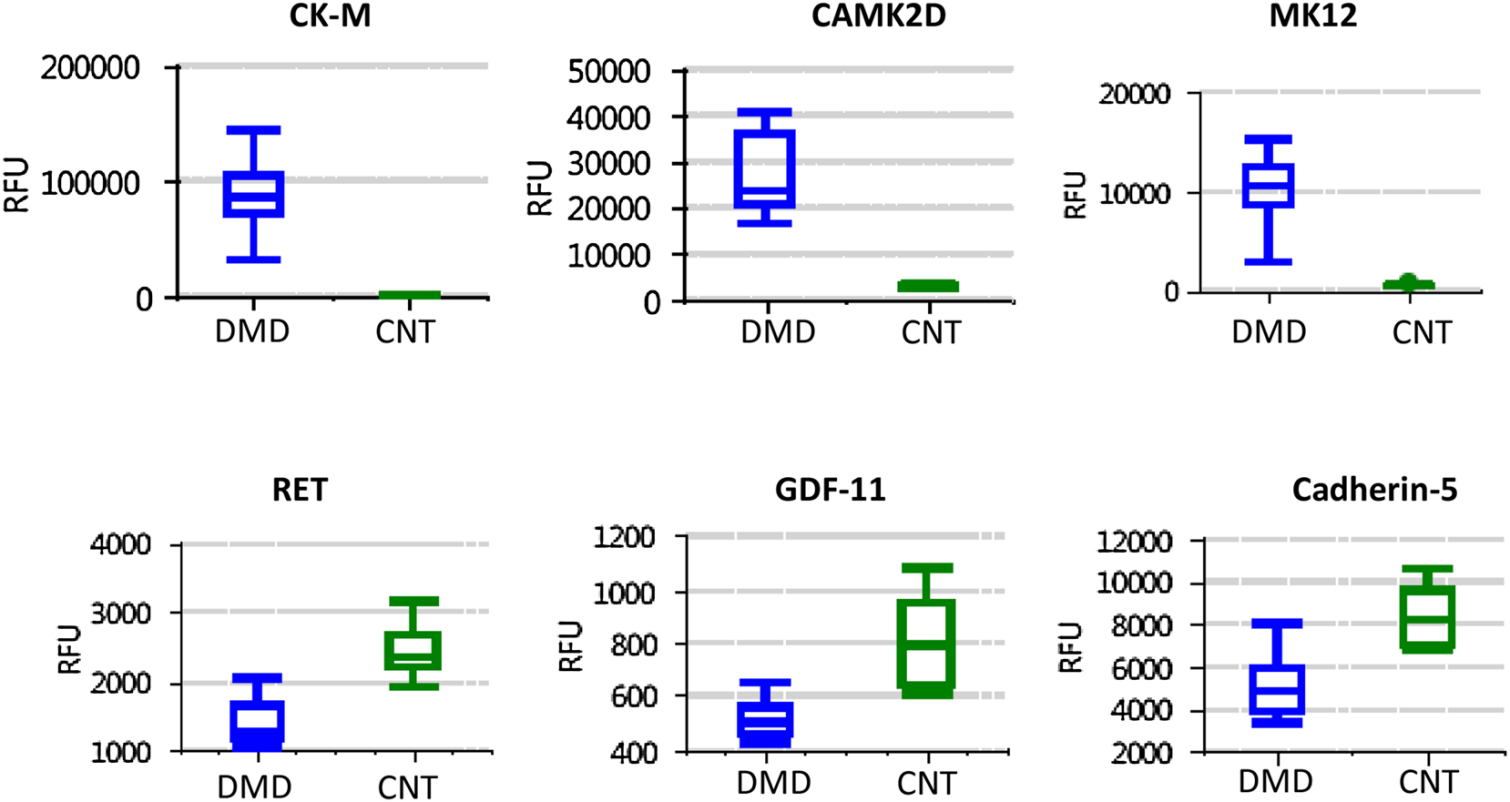
Representative serum protein biomarkers that are altered in 4 years old DMD boys. *CK-M* creatine kinase muscle type, *CAMK2D* calcium/ calmodulin-dependent protein kinase type II subunit delta; *MK12* mitogen-activated protein kinase 12, *RET* proto-oncogene tyrosine–protein kinase receptor Ret, *GDF-11* growth differentiation factor-11; Cadherin-5

 We and others have detected fragments of titin protein in serum and urine collected from very young DMD patients between the age 3 and 4 years old [44, 46, 47]. In our previous study titin fragments were detected by SDS-PAGE based mass spectrometry analysis in the mass range between 170 and 240 kDa [44]. These serum circulating titin fragments were further confirmed by Western blot analysis using titin monoclonal antibody# F146.9B9 from Enzo (Fig. 2). The titin fragment abundance decreases in older patients (≥10 years old), most likely due to a combination of loss of muscle mass and decreased activity. We hypothesis that the massive Ca^2+^ influx into muscle fibers during the early stage of the disease leads to disturbance of calpain activity and subsequent degradation of titin . This hypothesis is further supported by detection of N-terminal and C-terminal fragments of titin (potential calpain products) in urine samples from DMD patients as young as 3 years of age [48]. Furthermore, close examination of previously published SomaScan data [42] revealed additional markers that were significantly altered in their levels in younger DMD boys (~4 years of age) compared to age matched controls. As shown in Fig. 3, some of the biomarkers associated with DMD are already at their highest or lowest level in serum of 4 years old DMD boys compared to age matched controls. This suggests that muscle damage has already occurred at this young age, and perhaps even at birth. However, further biomarker studies in DMD infants (1–4 years old) are needed to confirm this hypothesis. If this is the case, then serum biomarker as an outcome measure would enable enrollment of younger DMD patients that have historically been excluded from clinical trials despite the fact that it is widely accepted that earlier treatment may have the largest impact on the disease.

## Identified DMD serum protein biomarkers are tied to specific pathobiochemical pathways and responsive to disease progression and age

Close examination of the protein biomarkers identified in the blood of DMD boys revealed four different groups of proteins that are tied to specific pathobiochemical pathways indicative of muscle fiber leakge, inflammation, fibrosis and muscle degeneration/regeneration. Table 1 summarizes these different groups of proteins and potential associated pathobiochemical pathways.

**Table 1:** DMD serum protein biomarkers are tied to specific pathobiochemical pathways

Group	Sub-group	Pathobiochemical pathway
Skeletal muscle enriched proteins (n = 21)	Myofibrilar proteins Glycolytic enzymes Ca^2+^ regulating proteins Transport proteins Other muscle enzymes	Membrane instability, leakage High levels in young DMD but then decreased with age. Unchanged in controls
Proteins involved in connective tissue remodeling (n = 14)	Cell adhesion proteins, connective tissue, proteases	Maintenance of extracellular matrix integrity All decreased in DMD versus controls
Inflammatory and immune system (n = 18)	Interleukins cytokine, chemokine	Inflammation and innate pathway Some increase with age in DMD Some decrease with age in DMD
Developmental processes (n = 6)	Cell differentiation Cell proliferation	Muscle regeneration Decreased with age in DMD while increased with age in controls

**Fig. 4:**
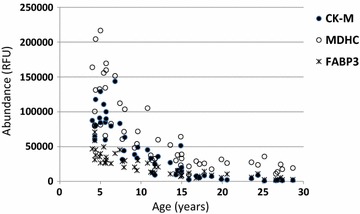
Plots showing changes in the serum level of three representative muscle leakage proteins in function of age in DMD patients. Data was generated using SomaScan proteome profiling on serum samples collected from 51 DMD patients with age ranging from 4 to 29 years old. Levels of the three proteins are plotted as relative fluorescent units (RFU) in function of age of DMD patients. *CK-M* creatine kinase muscle type, *MDHC* malate dehydrogenase cytosolic from, *FABP3* fatty acid binding protein 3

The first group of biomarkers, reflective of muscle fiber leakage, are skeletal muscle derived proteins and includes myofibrillar proteins, glycolytic enzymes, Ca^2+^ regulating proteins, transport proteins and other muscle specific enzymes. These proteins are all localized in the intracellular compartment of the muscle fiber and the release into the blood circulation is reflective of sarcolemma instability and leakage. These membrane leakage biomarkers were all highly elevated at young age (the youngest studied age was 4 years old) in blood of DMD patients relative to age matched healthy controls then gradually decreased with age in DMD patients while remained unchanged in controls [45], which is similar to CK-M. Figure 4 shows a correlation plot and changes in function of age of three representative proteins under this category (e.g. CK-M, malate dehydrogenase cytosolic (MDHC) and fatty acid binding protein-3 (FABP3). The decline in the level of these ‘CK–like’ biomarkers as a function of age reflects the progressive loss of muscle mass and disease progression in DMD patient [49] in agreement with earlier studies comparing levels of CK and pyruvate kinase in DMD patients across age [50].

**Fig. 5:**
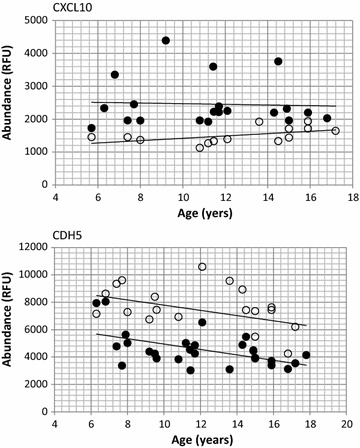
Plots showing changes in the serum level of two non-CK like biomarkers in function of age in DMD patients and healthy controls. Data was generated using SomaScan proteome profiling on serum samples collected from DMD patients (*filled circle*) and age matched healthy controls (*empty circle*). A plots is for a subset of DMD patients (n = 26) and age matched healthy controls (n = 20). *CDH5* cadherin-5, *CXCL10* C-X-C motif chemokine 10

The second group of biomarkers, reflective of fibrosis, consisted mostly of proteins involved in extracellular matrix remodeling and connective tissue organization. This group included hydrolases (e.g. disintegrin and metalloproteinase domain-containing protein 9 (ADAM9), phospholipase A2 (PLA2G2A), and cell adhesion proteins (e.g. cadherin-5; CDH5 and cell adhesion molecule 1; contactin-5). These proteins are localized mostly at the cell surface and in the extracellular compartment. The majority of cell adhesion proteins were lower in blood of DMD patients relative to controls at young age and remained lower throughout the age range, reflecting connective tissue disorganization. Proteases such metalloproteases were elevated in blood of DMD patients and might be involved in the extracellular matrix degradation. An example for cadherin 5 (CDH5) is shown in Fig. 5.

Third group of biomarkers, reflective of inflammation, consisted of 22 proteins involved in inflammatory and immune system processes and included mostly cytokines, chemokines, complements and interleukins. Some were elevated and some were decreased in serum of DMD patients compared to controls reflecting the inflammatory state and immune system disturbance in DMD. An example for C-X-C motif chemokine 10 (CXCL10) is shown in Fig. 5.

Finally the fourth group of biomarkers, reflective of muscle degeneration/regeneration, consisted of 6 proteins involved in developmental processes and included mostly cell differentiation and cell proliferation factors. The majority of these proteins were homeostatic in young DMD patients then decreased with age in DMD patients while increasing with age in healthy controls, probably reflecting the developmental disturbance of the skeletal muscle in DMD.

Biomarkers belonging to the last three groups are dissimilar to CK-M and reflect different pathogenesis progression of DMD.

Interestingly, some of the biomarkers that were found highly elevated in serum of DMD patients and mdx mice compared to their respective controls were found unchanged in their levels when comparing the proteome profiles of dystrophic muscle tissue to normal muscle tissue in the mdx mouse model except for titin, fatty acid binding protein 3, lactate dehydrogenase B (LDHB), and Troponin I fast skeletal muscle were found slightly decreased in dystrophic muscle relative to healthy muscle [51]. The slight decrease of FABP3 and LDHB was further confirmed by others in the soleus muscle of mdx mice relative to wild type mice [52]. The slight decrease of these proteins in muscle tissue and their dramatic increase in the blood circulation reflects a high cycles of muscle degeneration and regeneration in DMD in both human and animal models. Especially at younger age.

## Clinical utility of DMD circulating biomarkers

Efforts are under development to test the utility of DMD identified serum biomarkers in pre-clinical and clinical studies. Ideal biomarkers would enable acute, objective read outs of drug effect (pharmacodynamic biomarkers) and predict later benefit (surrogate biomarkers). However, to develop such biomarkers a good correlation between drug induced changes in the level of biomarkers and clinical benefit has to be established. A recent study has shown that myomesin-3, a membrane leakage biomarker, was sensitive to dystrophin replacement therapy in the mdx mouse model for DMD [48]. Myomesin-3 levels decreased toward normal levels in treated mice relative to untreated mice with correlation to restored dystrophin levels. Another more recent pre-clinical study performed on mdx-23 mouse model used the highly multiplexing SomaScan proteome profiling technology and identified multiple biomarkers that responded to dystrophin restoration in the mdx mouse model treated with peptide-antisense oligonucleotide conjugate [53]. Most of the responsive biomarkers were again associated with membrane leakage and included phosphoglycerate mutase 1, troponin I, Calcium/calmodulindependent protein kinase type II subunit beta (Camk2b), cytochrome-c and a disintegrin and metalloproteinase with thrombospondin motifs 5. Furthermore, several of the biomarkers detected by SomaScan technology and were found to be  elevated in the serumn of mdx mouse model relative to wild type mouse [53] were similar to those detect by the same technology to be elevated in DMD patients relative to controls [45]. To cite few, these included, CK-M, troponin-I, myoglobin, FABP3, LDHB, calcium/calmodulin dependent protein kinases, and cytochrome c. But few were unique to human samples and this is most probably due to the fact that aptamers were optimized to recognize human proteins and not mouse proteins. Nevertheless this technology can still be used in pre-clinical settings which will tremendously help testing the response of DMD biomarkers to new drugs in animal models. For instance the decrease in myoglobin, FABP3 and cytochrome c identified in mdx mouse model treated with PPMO [53] could be tested for their utility as pharcodynamic biomarkers to assess response to dystrophin replacement therapies in DMD patients.

In summary, several sarcolemma leakage biomarkers seem to be sensitive to dystrophin restoration. This suggests that restored (truncated) dystrophin is functional, stabilizes the sarcolemma and results in reduced leakage of muscle specific proteins into the blood circulation. These biomarkers must be validated and qualified for use as surrogate biomarkers in future clinical trials. At this time it is not known if reduction in biomarker levels is dose and time dependent. An ideal surrogate biomarker must be sensitive to early effects of the drug and predict later outcome. To answer these questions a carefully designed longitudinal experiment with different drug dosing are needed.

It is important to point out that each drug targeting specific pathobiochemical pathway in DMD (e.g. sarcolemma stabilization using dystrophin replacement therapies, reduction of muscle inflammation using glucocorticoids and analogue drugs, boosting muscle regeneration using growth factors) might affect a specific set of blood circulating biomarkers.

A recent study has shown that glucocorticoid treatment correlated with an increase in MMP9 levels in DMD patients [54]. However, an earlier study associated the increase of MMP9 with disease progression [40]. Currently there is an ongoing debate whether changes in MMP9 is associated with disease progression or response to glucocorticoid treatment [55]. But reliance on a single protein might not be ideal to distinguish changes due to disease progression or from a drug effect. In the list of DMD biomarkers we and others identified including miRNAs [31, 35, 41, 44–46] there is a large menu of biomarkers associated with different pathobiochemical pathways of muscle pathogenesis and disease stage. We believe a panel of biomarkers, rather than single biomarker, will allow better assessment of a drug effect. Furthermore, some drugs such as glucocorticoids might affect more than one pathobiochemical pathway to enhance muscle function as shown in a recent study conducted with low dose glucocorticoids in healthy subjects and mdx mouse model for DMD [56]. This further suggests the utility of a panel of biomarkers which might reflect the different pathways affected by a drug.

## Conclusions

Development of “omic” technologies has enabled discovery of novel biomarkers associated with DMD disease progression and response to therapies. Similar studies could be performed for a number of pediatric diseases that are in need of blood accessible molecular biomarkers to monitor disease progression and response to therapies. Defining robust and reliable serum pharmacodynamic biomarkers to monitor treatment in pediatric population will have an impact at several levels: (1) non-invasive and will minimize patient burden; (2) less subjective and can be used in most clinical trials; (3) Cost effective and will aid drug development programs.

## Additional file

10.1186/s12014-016-9109-x List of serum protein biomarkers indentified by mass spectrometry based proteome profiling and SomaScan technique References [44, 45].
